# Severe Pneumocystis jirovecii Pneumonia in an Apparently Immunocompetent Child Complicated by Acute Respiratory Distress Syndrome and Air Leak Syndrome: A Case Report and Literature Review

**DOI:** 10.7759/cureus.106357

**Published:** 2026-04-03

**Authors:** Ahmed Albishri, Mohaned Mohammed, Samah E Mohammed, Shady Wafa

**Affiliations:** 1 Pediatric Infectious Diseases, Armed Forces Hospital Southern Region, Khamis Mushait, SAU; 2 Pediatric Medicine, Armed Forces Hospital Southern Region, Khamis Mushait, SAU; 3 Pediatrics, Armed Forces Hospital Southern Region, Khamis Mushait, SAU

**Keywords:** ards in children, immunocompetent patients, pediatric pneumonia, pediatric pneumothorax, pneumocystis jirovecii pneumonia (pjp)

## Abstract

*Pneumocystis jirovecii* pneumonia (PJP) is an opportunistic fungal infection that predominantly affects immunocompromised individuals. Reports in immunocompetent children are rare. We report the case of a previously healthy 5½‑year‑old Saudi girl who presented with fever, dry cough, and progressive respiratory distress. Her condition rapidly deteriorated, requiring pediatric intensive care admission and mechanical ventilation due to severe acute respiratory distress syndrome. Her course was complicated by pneumothorax, pneumomediastinum, and extensive surgical emphysema. Extensive microbiological investigations were negative except for bronchoalveolar lavage demonstrating *Pneumocystis jirovecii* using Gomori methenamine silver staining. The patient was treated with intravenous trimethoprim-sulfamethoxazole and supportive intensive care management with gradual clinical recovery. Immunological evaluation, including lymphocyte subsets, immunoglobulin levels, complement levels, vaccine antibody responses, and whole‑exome sequencing, revealed no evidence of primary or secondary immunodeficiency. Long‑term follow‑up for more than 10 years showed no recurrent severe infections. This case highlights that PJP may rarely occur in immunocompetent children and can present with life‑threatening respiratory failure. Awareness of this possibility is important in severe unexplained pneumonia.

## Introduction

*Pneumocystis jirovecii* pneumonia (PJP) is a life‑threatening opportunistic infection caused by the fungal organism *Pneumocystis jirovecii* [1]. It is most commonly associated with impaired cellular immunity, particularly in patients with human immunodeficiency virus (HIV) infection, hematologic malignancies, organ transplantation, and prolonged corticosteroid therapy. Before the initiation of prophylaxis, it was a leading cause of infectious mortality in pediatric leukemia patients [2]. Molecular analyses, including ribosomal RNA sequencing and mitochondrial DNA studies, have demonstrated that this organism is classified as a fungus [3]. *Pneumocystis jirovecii* belongs to the genus *Pneumocystis* and is a human-specific opportunistic fungal pathogen [4]. In immunocompetent individuals, *Pneumocystis jirovecii* infection is typically asymptomatic or causes mild self-limited disease; however, PJP predominantly occurs in severely immunocompromised hosts [5]. In pediatric populations, the infection is typically linked to primary immunodeficiency disorders or immunosuppressive therapy. Nevertheless, extremely rare cases have been reported in patients without identifiable immunodeficiency. The mechanisms underlying these infections remain poorly understood but may include transient immune dysregulation, high environmental exposure, or unrecognized subtle immune defects [6]. Here, we describe a severe case of PJP in a previously healthy child who developed acute respiratory distress syndrome (ARDS) and air leak complications, yet demonstrated no evidence of underlying immunodeficiency even after extensive immunological and genetic evaluation. Favorable outcomes of PJP in immunocompetent patients can be achieved through early recognition and prompt therapeutic intervention.

## Case presentation

A previously healthy 5½‑year‑old Saudi female presented with a three‑day history of high‑grade fever, dry cough, progressive shortness of breath, and reduced oral intake. She had recent contact with a sibling who had an upper respiratory tract infection. There was no history of chronic illness, recurrent infections, or immunodeficiency in the patient or family.

**Figure 1:**
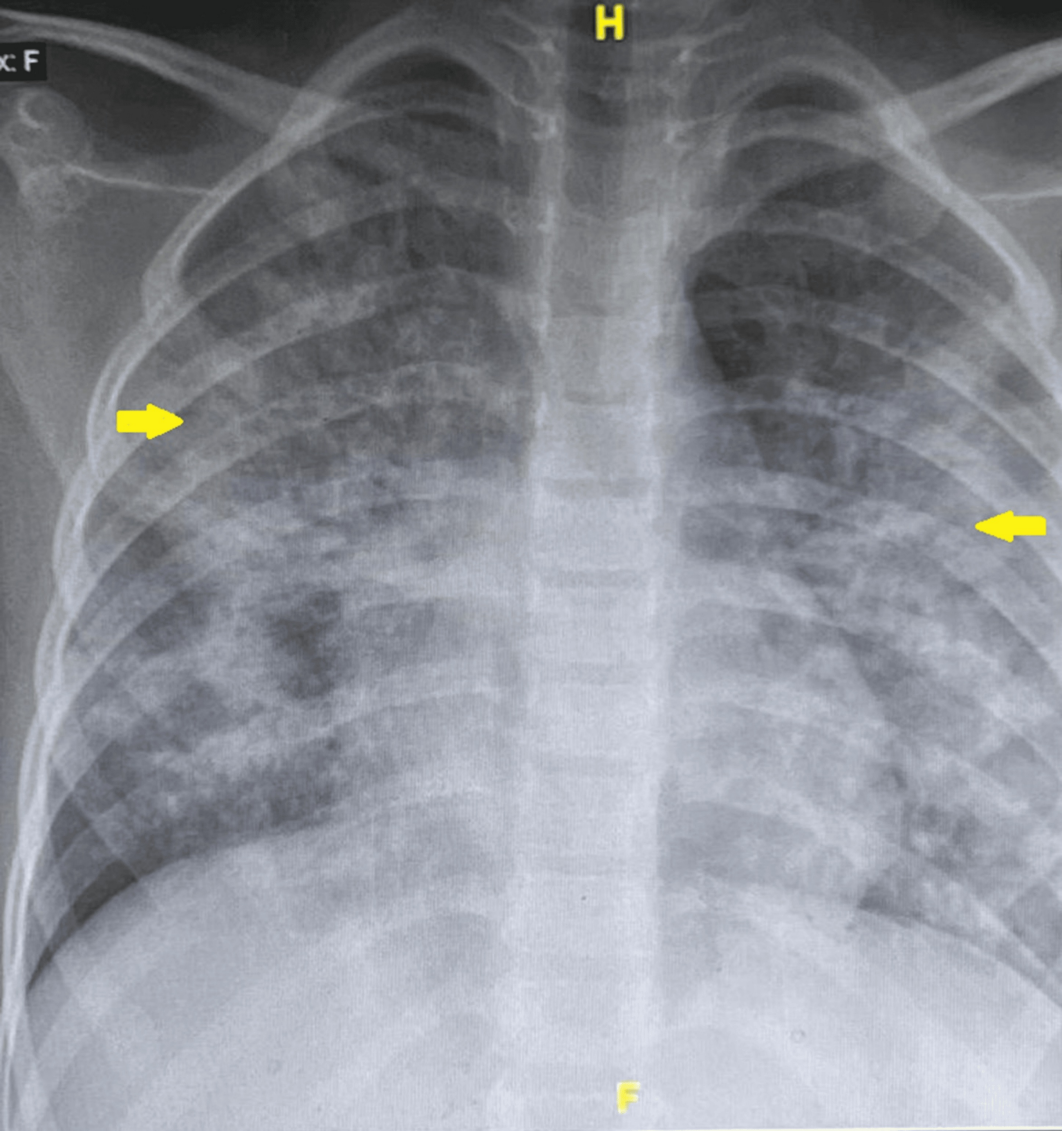
Chest X-ray at the time of admission. Chest radiography demonstrated bilateral non‑homogeneous opacities involving the middle and lower lung zones, as demonstrated by the yellow arrow.

On admission, the patient appeared ill and in significant respiratory distress. Vital signs included a temperature of 39.2°C, a heart rate of 102 beats/minute, a respiratory rate of 50 breaths/minute, blood pressure of 100/60 mmHg, and oxygen saturation of 80-84% on room air. Chest radiography demonstrated bilateral non‑homogeneous opacities involving the middle and lower lung zones (Figure 1). Laboratory investigations revealed leukopenia with elevated inflammatory markers (Table 1).

**Table 1:** Initial laboratory investigations during admission showing leukopenia with elevated inflammatory markers. TWBC = total white blood cell count; CRP = C-reactive protein; PLT = platelets; LDH = lactate dehydrogenase; PCR = polymerase chain reaction; HIV = human immunodeficiency virus

Description	Result	Unit	Reference range
TWBC	3.1	10^9^/L	4.5–13.5
Hemoglobin	12.5	g/dL	10.9–15
PLT	246	10^9^/L	150–450
CRP	52	mg/L	<1
LDH	497	mmol/L	155-290
*Mycoplasma* PCR (nasopharyngeal swab)	Negative		
Acid-fast bacilli (bronchoalveolar lavage)	Negative		
Blood culture	Negative		
HIV Ag/Ab	Negative		
*Chlamydia pneumoniae* (nasopharyngeal swab)	Negative		

**Figure 2:**
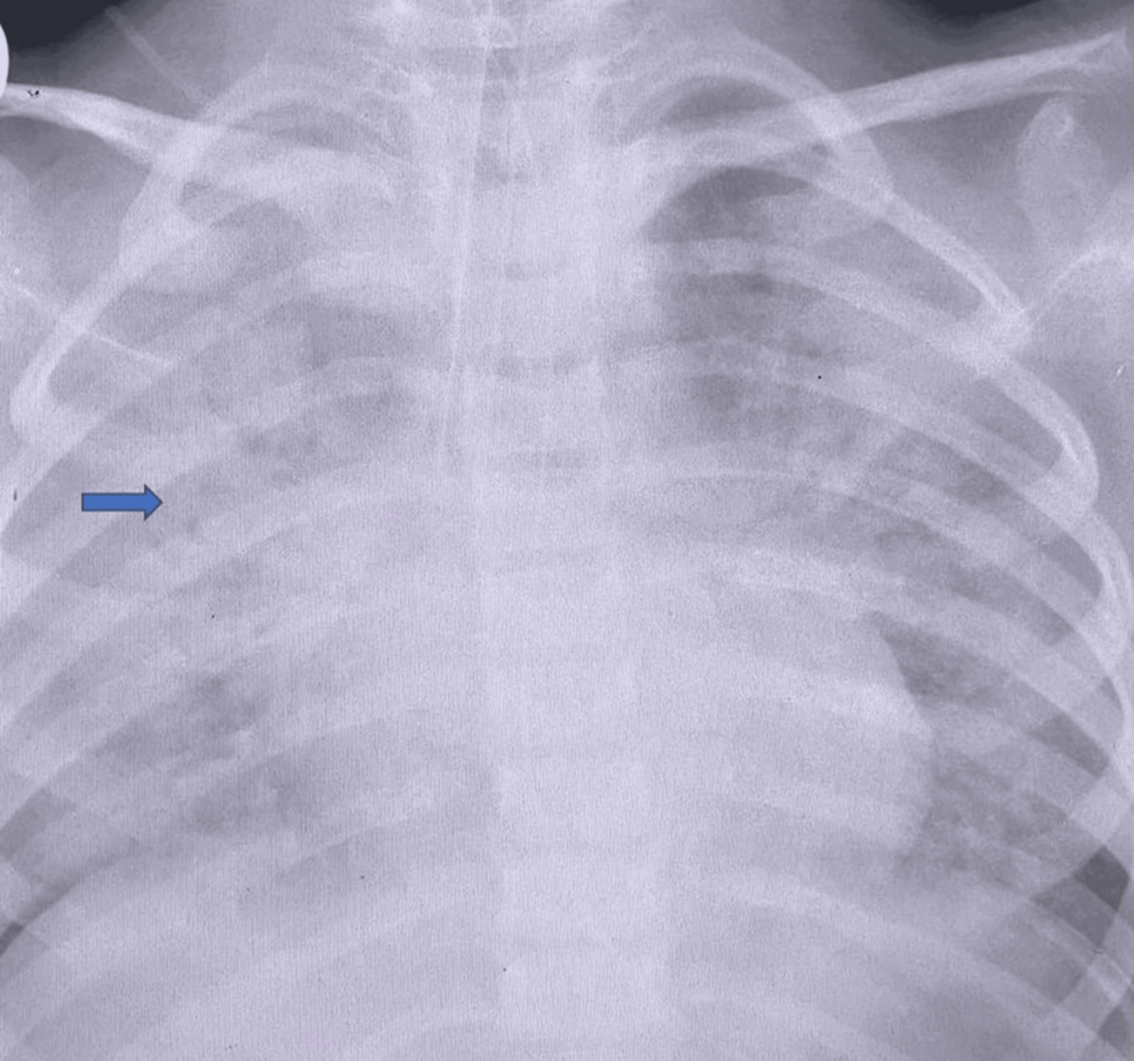
Chest X-ray showing acute respiratory distress syndrome. Chest X-ray showing acute respiratory distress syndrome after three days of admission upon admission to the intensive care unit, as demonstrated by the blue arrow.

**Figure 3:**
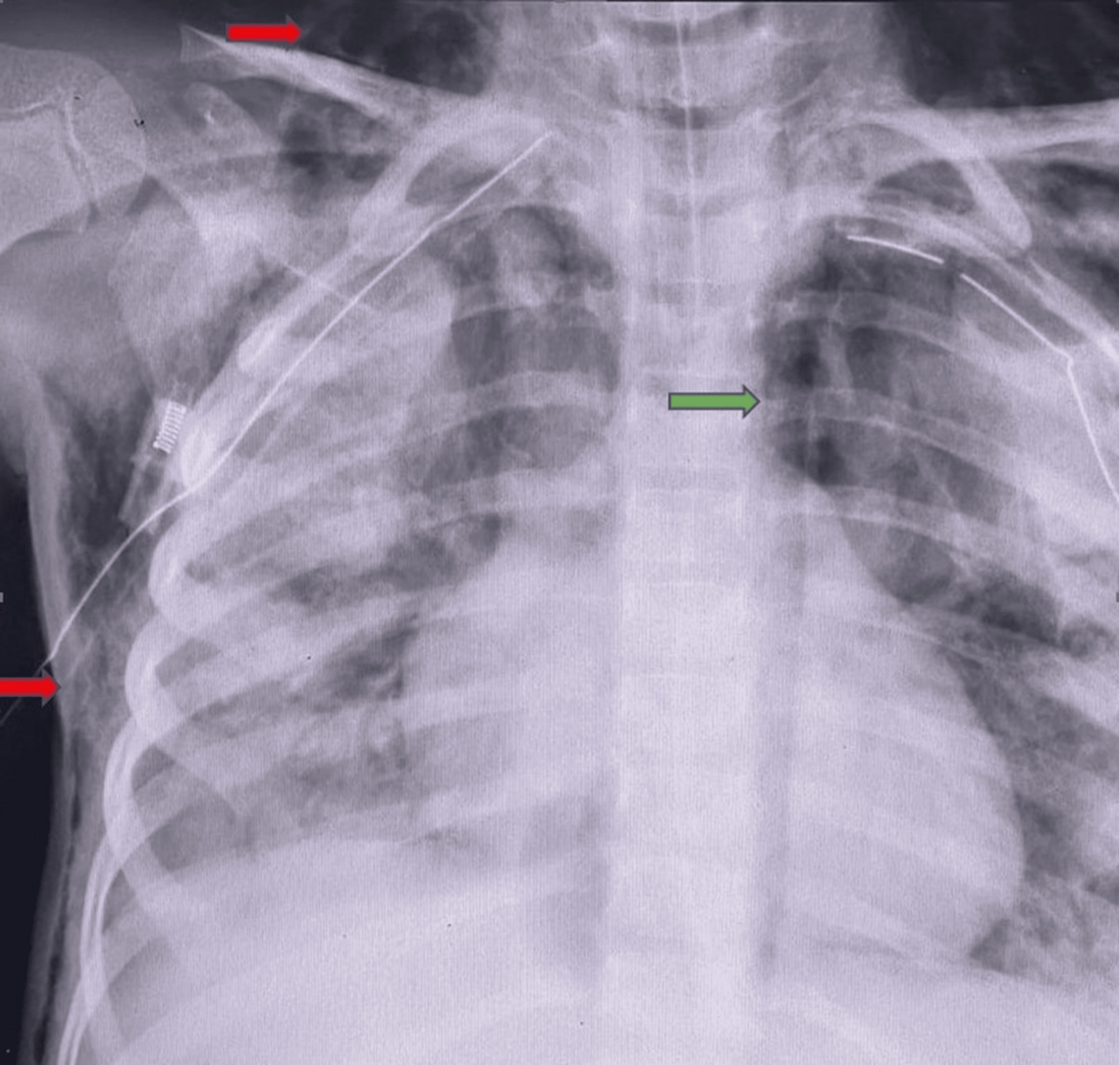
Chest X-ray after complication with air leak syndrome. Chest X-ray showing pneumothorax (green arrow) requiring chest tube insertion and extensive surgical emphysema (red arrow).

Despite treatment with intravenous cefuroxime and supportive oxygen therapy, her respiratory condition deteriorated. On the third hospital day, she developed worsening hypoxemia and respiratory acidosis, necessitating transfer to the pediatric intensive care unit, where she was intubated and mechanically ventilated. Her clinical course was complicated by ARDS (Figure 2), pneumothorax requiring chest tube insertion, pneumomediastinum, and extensive surgical emphysema (Figure 3). She was managed with high-frequency oscillatory ventilation, inhaled nitric oxide, methylprednisolone, and surfactant (Survanta). Antibiotic therapy was escalated to vancomycin, meropenem, and azithromycin.

Extensive microbiological investigations, including bacterial cultures and atypical pathogen testing, were negative except for bronchoalveolar lavage, which demonstrated *Pneumocystis jirovecii* using Gomori methenamine silver staining. Viral respiratory multiplex polymerase chain reaction (PCR) showed human metapneumovirus with negative results for other viruses. Human metapneumovirus may act as a predisposing factor or coexist with severe respiratory infections such as PJP, like our patient, particularly in immunocompromised individuals, as viral respiratory infections may contribute to pulmonary epithelial damage and immune modulation, potentially facilitating opportunistic infections. Intravenous trimethoprim-sulfamethoxazole therapy was initiated with gradual clinical improvement. The patient required mechanical ventilation for nine days before successful extubation. She completed a 21‑day course of therapy and was discharged in stable condition. Comprehensive immunological evaluation (Table 2), including immunoglobulin levels, complement levels, lymphocyte subsets, vaccine antibody responses, and whole‑exome sequencing, showed no evidence of immunodeficiency. Long‑term follow‑up over the subsequent decade revealed normal health with no recurrent severe infections. The patient has been followed regularly in the pediatric infectious disease clinic. She is currently 16 years old, with a normal life with no recurrent severe infections or evidence of underlying immunodeficiency.

**Table 2:** Immunological evaluation showing no evidence of immunodeficiency. IgG = immunoglobulin G; IgA = immunoglobulin A; IgM = immunoglobulin M; IgE = immunoglobulin E; C3 = complement 3; C4 = complement 4; CD = cluster of differentiation

Description	Result	Unit	Reference range
IgG	7.32	g/L	6–13
IgA	0.699	g/L	0.6–2.2
IgM	0.544	g/L	0.4–1.6
IgE	51.1	IU/ml	<1
C3	1.05	g/L	0.9–1.8
C4	0.19	g/L	0.16–0.47
T-lymphocyte subsets			
Absolute lymphocyte count	2,200	µL	1,100–5,900
T-cell (CD3+)	1478	µL	700–4,200
T-helper cells (CD3+/CD4+)	43.7	%	31–47
T-cytotoxic cells (CD3+-CD8+)	22.1	%	18–35
B cells (CD19)	21.5	%	13–27
CD4:CD8 ratio	1.97	%	More than 1.0
Vaccine antibody responses	Tetanus IgG: 0.8 IU/mL (normal: 0.3 IU/mL), pneumococcal antibodies: 52.7 mg/L (adequate response; reference ≤3.3 mg/L)		
Whole-exome sequencing	Analysis did not reveal any pathogenic or likely pathogenic variants associated with primary immunodeficiency disorders		

## Discussion

PJP is traditionally regarded as an opportunistic infection occurring in patients with impaired cellular immunity. However, an increasing number of reports have documented infection in individuals without identifiable immunodeficiency. In children, most cases are associated with primary immunodeficiency disorders or immunosuppressive therapy [2]. Nevertheless, extremely rare cases in previously healthy children have been reported. These cases raise important questions regarding host susceptibility and the possibility of transient immune dysfunction or environmental exposure to colonized carriers.

The present case is notable for several reasons. First, the patient had no prior history of recurrent infections, and extensive immunological evaluation failed to demonstrate any immune abnormality. Second, the disease manifested with a severe clinical course, including ARDS and air leak syndrome, complications that are more frequently reported in non‑HIV PJP cases. Third, long‑term follow‑up confirmed the absence of recurrent infections, supporting the conclusion that the infection occurred in a truly immunocompetent host. PJP in non-HIV immunocompetent hosts is typically characterized by rapidly progressive dyspnea and a more acute and severe clinical course compared with HIV-infected patients. It is often associated with a poorer prognosis and higher mortality rates.

The diagnosis of PJP is based on a combination of clinical and radiological suspicion, assessment of patient risk factors, and microbiological confirmation through induced sputum examination, bronchoalveolar lavage analysis, or, when necessary, lung biopsy [7]. The exact source and reservoir of infection have not yet been clearly identified, making the understanding of the transmission dynamics and epidemiology of PJP infection an ongoing challenge [8]. It is believed to arise either from a newly acquired (de novo) infection or from reactivation of a latent infection acquired during childhood [9]. To our knowledge, only one published case has described PJP in an immunocompetent female pediatric patient aged six months, admitted to Virgen del Rocío University Hospital with the same presentation as our case, with a history of a non-productive cough, difficulty breathing, and a negative family history for immunodeficiency or HIV infection. *Pneumocystis jirovecii* DNA was detected in nasopharyngeal aspirate samples by amplifying the mitochondrial large-subunit gene of rRNA with nested PCR. No other infections were detected by culture, molecular tests, or serologic tests. The infant was treated with high-dose trimethoprim-sulfamethoxazole and adjuvant steroids. She did well and was discharged a month later [10]. Most reported cases in the literature involve individuals with underlying immunodeficiency, those receiving immunosuppressive therapy, or adults taking medications that impair immune function [11]. This makes the present case particularly rare and noteworthy.

## Conclusions

This case highlights that PJP may rarely occur in immunocompetent children and can present with life‑threatening respiratory failure. Awareness of this possibility is important in severe unexplained pneumonia. Clinicians should maintain a high index of suspicion for PJP even in previously healthy patients when clinical presentation and radiological findings are suggestive of the disease. In such cases, prompt diagnostic evaluation and early initiation of appropriate anti-*Pneumocystis* therapy should not be delayed pending confirmation of immune status.
